# How Do Team Cooperative Goals Influence Thriving at Work: The Mediating Role of Team Time Consensus

**DOI:** 10.3390/ijerph19095431

**Published:** 2022-04-29

**Authors:** Mingze Li, Shuting Peng, Liwen Liu

**Affiliations:** 1 School of Management, Wuhan University of Technology, Wuhan 430070, China; mingze@whut.edu.cn (M.L.); pengshuting@whut.edu.cn (S.P.); 2 School of Politics & Public Administration, Soochow University, Suzhou 215123, China

**Keywords:** team cooperative goals, team temporal leadership, team time consensus, team thriving at work

## Abstract

Thriving at work is beneficial to the physical and mental health of individuals, promotes the innovation and development of organizations, and is a shield against job burnout. However, the current research on the antecedents of thriving at work lacks the exploration of team characteristics. This study introduces team temporal leadership as a moderating variable and team time consensus as a mediating variable to explore the relationship between team cooperative goals and thriving at work. Based on the analysis of 326 data from 92 teams, the results showed that: (1) Team cooperative goals have a significant positive impact on team time consensus, and team time consensus has a significant positive impact on team thriving at work; (2) Team time consensus mediates the relationship between team cooperative goals and team thriving at work; and (3) Team temporal leadership not only moderated the relationship between team cooperative goals and team time consensus, but also moderated the indirect effect of team cooperative goals on team thriving at work through team time consensus. This study enriches the research on the triggering mechanism of thriving at work to some extent and provides enlightenment for organizations to stimulate the state of thriving at work.

## 1. Introduction

Thriving at work is a psychological state in which an individual experiences both “vigor” and “learning” while working [1], which has important implications for both individuals and organizations. Existing studies have confirmed that thriving at work can alleviate burnout, improve physical and mental health [2], and promote personal development [3]. It can also stimulate employees’ innovative vitality [4] and improve work performance [2]. On the whole, it is beneficial to improve the performance of the organization and promote the innovation of the organization [5]. A joint survey shows that 70% of employees experience job burnout. On-the-job employees tend to lose enthusiasm, lack motivation for work, and reduce work engagement. Because of heavy work tasks and long-term work pressure, job burnout has become a common problem in people’s workplace [6,7]. It seriously damages people’s physical and mental health and hinders the long-term development of teams and organizations [8]. Therefore, how to keep team members thriving at work is a problem that managers in organizations are very concerned about.

Existing research mainly studies the emergence of thriving at work from the perspectives of work resources, unit contextual features, leadership styles, and personal characteristics [9,10,11,12]. Most of these factors emphasize that human-to-human and human–environment interactions contribute to thriving at work, such as decision-making discretion, broad information sharing, and climate of trust and respect [1,5], but have a lack of attention to the characteristics of team orientation and team task itself, such as team goals. Deutsch (1949) in his cooperation and competition theory argues that people’s beliefs about how their goals are related determines the way they interact, which in turn affects their performance and team cohesion [13]. According to cooperation and competition theory, team goals have three forms: cooperation, competition, and independence. The cooperative goals have a positive impact on the team. Research has confirmed that team cooperative goals create a good atmosphere of initiative and innovation for team members, making team members believe that they have team potential to complete a range of tasks. This motivates team members to persist in overcoming obstacles and take initiative to innovate [14]. The cooperative goal is indeed inspiring to employees [15], making employees believe that the team is a community of interests, and making employees more motivated to display their talents and take responsibility [16]. Therefore, it is necessary to explore the connection and mechanism between team cooperative goals and thriving at work.

Regarding the mediating mechanism of thriving at work, the socially embedded model believes that unit contextual features and work resource factors indirectly influence thriving at work through individuals’ agentic work behaviors [1]. An integrative model of human growth at work points out that the unit contextual features can use employee psychological factors as a medium to affect thriving at work, such as autonomy, competence, and sense of belonging [17]. Recent studies have focused on the mediating mechanism of leadership style on thriving at work, such as psychological empowerment [18], relational energy [19], and leader–member exchange [20]. However, current research ignores the temporal perspective. In reality, individuals in teams generally have temporal differences and diversity [21]. Mismatches and inconsistencies in team members’ temporal perspectives may lead to team conflicts, or may hinder the team’s ability to effectively coordinate individual actions [22], which can have an impact on team members and overall team performance, so the temporal perspective deserves attention. Based on temporal perspective, this study introduces team time consensus as a mediating variable and team temporal leadership as a moderating variable to explore the link between team cooperative goals and team thriving at work.

## 2. Theory and Hypothesis

### 2.1. Team Cooperative Goals and Team Time Consensus

According to the theory of cooperation and competition, team goals have three forms: cooperation, competition, and independence. Team cooperative goals refer to the degree to which the goals of team members are positively related [13,23]. Team members with cooperative goals believe that their goals are positively related, the achievement of other members’ goals contribute to their own goals, and that the members of the team as a whole work together. Team members with cooperative goals perceive themselves as being in a “win-win” relationship [24], which facilitates team time consensus.

First, existing studies have pointed out that team cooperative goals emphasize the consistency of team goals and enhance the team identification [16]. A high level of team identification strongly correlated their self-esteem and sense of self-worth with team success and failure [25]. Driven by such team values, team members are willing to discuss conflicting issues with an open mind and in a collaborative manner, with the aim of seeking solutions that benefit all team members [26] and promoting team members’ willingness to communicate with each other to reach a consensus. In addition, team cooperative goals can allow team members to reduce the vicious perception of conflict, view conflict as a common problem that needs to be solved cooperatively, and reduce conflict [27,28]. Although individuals may have different views and arrangements for time, the team cooperative goals will allow individuals to make concessions for the common goals of the team, reduce conflicts, increase coordination among members, and tend to form team time consensus. Thus:

**Hypothesis** **1** **(H1).** *Team cooperative goals positively affect team time consensus*.

### 2.2. Team Time Consensus and Thriving at Work

In real life, team members generally have individual differences in pacing style, time urgency, and time perspective [29]. This difference often leads to time cognitive conflicts, frustrates employees’ work enthusiasm, triggers negative emotions, and affects work performance [30]. However, team time consensus is an important implicit time coordination mechanism, representing a consistent understanding among team members on task schedules, cycles, and deadlines, which facilitates the generation of tacit time understanding among members [21,31]. According to the socially embedded model [1], unit contextual features (decision-making discretion, broad information sharing, and climate of trust and respect) and work resources (knowledge, positive meaning, positive affective resources, and relational resources) are the ante-factors of thriving at work, and team time consensus can promote team thriving at work.

From the perspective of unit contextual features, team time consensus can create a good working atmosphere for the team. Team time consensus can reduce differences and conflicts, create an atmosphere of harmony and unity, and promote mutual learning among team members [32]. From the perspective of work resources, after the team time consensus is formed, it is beneficial to reduce the cognitive resources consumed by the running-in of the team task time frame, focus resources on team tasks, and stimulate team creativity [30]. Team time consensus is also beneficial to promote the smooth progress of work and effective collaboration. Team members can meet deadlines through better coordinated actions, resulting in positive team performance [21,31], bringing better opportunities and resources to the team, enhancing team self-confidence and work meaningfulness, and stimulating team vitality. Thus:

**Hypothesis** **2** **(H2).** *Team time consensus will positively affect team thriving at work*.

### 2.3. The Mediating Role of Team Time Consensus

Team time consensus is a shared understanding of the time aspects of completing tasks such as task progress, pace of work activities, and deadlines [21,31]. Cooperative goals among team members can promote positive expectations, resource exchanges, and open-minded discussions among team members [33,34]. Team members with cooperative goals will be willing to make individual concessions for the achievement of the team’s overall goals, and agree on the timing of team tasks and the rhythm pace of task progress. Therefore, team members with team cooperative goals, expecting to perform effectively with each other, will interact in a way that promotes common goals and mutually beneficial problem solving [24], which facilitates consensus among team members in terms of time. The team time consensus can reduce the ambiguity and uncertainty of time and increase the likelihood that group members can coordinate their actions effectively [21]. Thus, team time consensus is conducive to further form a working atmosphere of common advancement and retreat, promote the willingness to learn from each other, stimulate the vitality of the team, and facilitate the emergence of team thriving at work. Therefore, combining hypotheses H1 and H2, this study proposes the following hypothesis:

**Hypothesis** **3** **(H3).** *Team time consensus mediates the relationship between team cooperative goals and team thriving at work*.

### 2.4. The Moderating Role of Team Temporal Leadership

Team temporal leadership is a leader behavior that helps build, coordinate, and manage the speed of task completion in a team [35]. Effective team temporal leadership can help team members schedule activities, unify the rhythm pace of activities, minimize time conflicts between members and tasks, and promote consensus among members in terms of time allocation, thereby enabling the team to form shared temporal cognitions [36]. Temporal leadership is the driving force that promotes the formation of team time consensus [37].

First, through a unified time plan, such as formulating a periodic table of tasks, temporal leadership weakens the heterogeneity of individuals in time focus, promotes the time focus of team members [38], and helps team members reach consensus on time. Second, team temporal leadership can incorporate diverse individual time characteristics into the team consensus track through time management to form a unified team time rhythm [30], which is conducive to the formation of time consensus. Finally, the development of time consensus is facilitated by temporal leadership involving time planning in the early stages of the project and time reminders in the later stages of the project [31]. In addition, temporal leadership can reduce the negative emotions brought about by time pressure, improve the psychological state of employees [39], and improve happiness [40]. When employees are in a better emotional state, they are more likely to cooperate and reach a consensus on time.

Therefore, the level of team temporal leadership may affect the relationship between team cooperative goals and time consensus. When team temporal leadership is high, the differences and diversity of individuals’ time perspectives are buffered [36], and the positive relationship between team cooperative goals and team time consensus is more significant. Thus:

**Hypothesis** **4a** **(H4a).** *Team temporal leadership moderates the relationship between team cooperative goals and team time consensus*.

Combined with H3 and H4a, a moderated mediating model is proposed. The effect of team cooperative goals on team thriving at work through team time consensus is enhanced when temporal leadership is higher. Under high-level team temporal leadership, the leader schedules team time resources, controls the progress of team tasks, integrates team time diversity into a unified track, reduces time conflicts and differences, and reaches team time consensus [30]. At this time, the working atmosphere of the team is good, and the members cooperate with each other, so as to promote the thriving at work of the whole team. Thus:

**Hypothesis** **4b** **(H4b).** *Team temporal leadership moderates the mediating effect of team time consensus between team cooperative goals and team thriving at work. The mediating effect of team time consensus is stronger when team temporal leadership is at a high level*.

**Figure 1:**
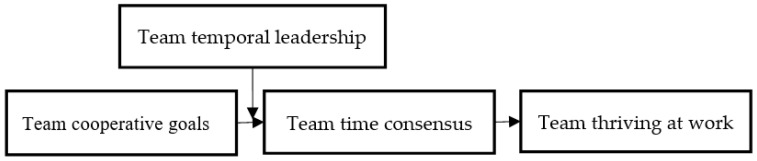
Theoretical Model.

In summary, the theoretical model of this study is shown in Figure 1. 

## 3. Method

### 3.1. Participants and Procedure

This research adopts the method of questionnaire survey. Before the study began, we consulted with local electronics companies whether they would like to participate in our survey, and got a positive response from some companies. After synthetically considering both the geographical and size of the company, we finally selected four larger electronics companies from four different cities of Hangzhou, Shanghai, Shenzhen, and Chengdu. The survey be conducted in April 2021. The main reason for selecting this industry is that the electronic technology industry has high requirements for employees’ rapid response and creativity, and requires employees to maintain a state of full of energy and continuous learning. In addition, our questionnaire was filled out by teams (team leaders and employees filled out different questionnaires), and the team size was limited to 3–5 people. Employees of electronic companies usually work in teams, such as numerous product research and development teams. This is consistent with our survey.

The researchers first got in touch with the management of the company, and after the approval of the management, they consulted with the person in charge of the human resources department to obtain a list of employees participating in this survey. Next, the researchers grouped various departments and teams according to the list, each with 3–5 employees in the team, and numbered them so that the later team leaders could match the questionnaires of the team employees.

After negotiating the survey time period with the company contact, the researchers came to the site to distribute the questionnaires. Before filling out the questionnaire, the researcher first explained to the respondents who participated in the questionnaire that the questionnaire answering followed the principle of complete voluntary, promised the confidentiality of the research data, and explained the precautions for filling out the questionnaire. This research adopts the method of matching between leaders and employees, in which employees evaluate demographic information, team cooperative goals, team temporal leadership, and team time consensus; the content of leaders evaluation is employee thriving at work.

In this study, a total of 500 questionnaires were sent to 104 teams, of which 95 teams’ questionnaires were recovered, with a total of 438 data, and the questionnaire recovery rate was 87.6%. Excluding invalid samples with unanswered questions, the final valid samples were 326 questionnaires from 92 teams, and the valid filling rate was 65.2%. Among them, the age mainly concentrated in the 25–40 years old group, accounting for 75.4%, the oldest is 60 years old; The proportion of male and female is 43.9% and 56.1%, showing a balanced gender ratio. The proportion of those with high school degrees or below is 23.6%, those with junior college degrees are 15.2%, those with bachelor’s degrees are 52.8%, and those with master’s degrees are 8.4%. Years of working include new employees who have been on the job for a few months and old employees who have been working for 37 years; The years of working with leaders are mainly 1–5 years, accounting for 64.3%. The shortest period of working with leaders is 3 months, and the longest is 17 years.

### 3.2. Questionnaire Design and Variable Measurement

The questionnaire of this survey is mainly composed of the following parts, the first part is the introduction to the questionnaire, the second part is the specific questions of variable measurement, and the third part is demographic information (including: gender, age, education level, working years and years of working with leaders). Among them, the questions are derived from the mature scale developed and verified by previous scholars, which has good reliability and validity. The study was scored on a five-point Likert scale (1 = strongly disagree to 5 = strongly agree). The specific variable measurements are as follows: 

Team cooperative goals: We measured team cooperative goals using five-item scale developed by Alper et al. (1998) [33]. Tjosvold et al. (2004) also used this scale in their research (α = 0.82) [41]. Team members were asked to rate these items based on their own feelings about their team. A sample item was “When our team members work together, we usually have common goals.” The reliability of the scale is α = 0.855 and aggregations were justified (mean Rwg(j) = 0.933; ICC (1) = 0.411, ICC (2) = 0.712). 

Team temporal leadership: We measured team temporal leadership using seven-item scale developed by Mohammed and Nadkarn (2011, α = 0.90) [35]. Maruping et al. (2015) also used this scale in their research (α = 0.77) [39]. Team members were asked to rate these items based on their own feelings about their team leader behavior. A sample item was “My leader urge members to finish subtasks on time”. The reliability of the scale is α = 0.892 and aggregations were justified (mean Rwg(j) = 0.87; ICC (1) = 0.407, ICC (2) = 0.708).

Team time consensus: We measured team time consensus using four-item scale developed by Gevers et al. (2006) [21]. Gevers et al. (2009) also used this scale in their research (α = 0.74) [31]. Team members were asked to rate these items based on their own feelings about their team. A sample item was “Our team members have the same opinions about meeting deadlines”. The reliability of the scale is α = 0.842 and aggregations were justified (mean Rwg(j) = 0.933; ICC (1) = 0.415, ICC (2) = 0.715).

Thriving at work: We measured thriving at work using ten-item scale developed by Porath et al. (2012) [10]. The scale has two dimensions (Learning and Vitality). Walumbwa et al. (2018) also used this scale in their research (α = 0.88) [12]. Leaders were asked to rate these items based on their team members’ performance and behavior in work. Sample items include “This employee continue to learn more and more as time goes by” and “This employee have energy and spirit”. The reliability of the scale is α = 0.769 and aggregations were justified (mean Rwg(j) = 0.982; ICC (1) = 0.717, ICC (2) = 0.9).

Data Aggregation. We aggregated individual level ratings of team cooperative goals, team temporal leadership, team time consensus, and thriving at work by group members to the team level of analyses. Therefore, it is necessary to examine whether individual-level data can be aggregated to the team level from the aspects of within-group consensus and within-group reliability [42,43]. Commonly used metrics are Rwg, ICC (1), and ICC (2). Referring to the research of previous scholars, RWG is judged to be greater than 0.7 [44,45], ICC (1) is judged to be greater than 0.12 [46], and ICC (2) is judged to be greater than 0.47 [43]. The calculation results of the team cooperative goals, team temporal leadership, team time consensus, and thriving at work are shown above, and all meet the criteria for judging.

Control variables: Drawing on the selection of control variables by Walumbwa et al. (2016) [47], this study uses the average team age, gender, education level, years of working, and years of working with leaders as control variables.

### 3.3. Data Analysis

Descriptive analyses and correlation analyses were performed using SPSS 24.0 and regression analyses were conducted using Mplus version 8.0. For the mediation effect hypothesis, the deviation-corrected parameter Monte Carlo [48] was used to estimate the mediation effect and test the significance. For the moderation effect, in order to avoid multicollinearity, this study centralizes the independent variables before constructing the interaction terms, and uses simple slope analysis [49] to explore the slope significance of the adjustment variables under different values (mean plus minus one standard deviation). For the moderated mediation effect, we adopted the Monte Carlo simulation and constructed confidence intervals (CIs) to test the indirect effects at high (1 SD above) and low (1 SD below) levels of the moderator [50].

## 4. Result

We tested the discriminant validity of the focal scales with confirmatory factor analysis (CFA). Drawing on the parceling method used by Qin et al. (2020) [51], we created parcels of items for the thriving at work and team temporal leadership constructs. Table 1 shows the fit indices of the CFA. The hypothesized four-factor model demonstrated good fit (χ^2^/Df = 4.826, CFI = 0.897, TLI = 0.864, SRMR = 0.054), and fit the data better than any of the models (see Table 1).

Descriptive statistics and correlations are presented in Table 2. The results preliminarily support our hypothesis.

The unstandardized coefficients for the hypotheses test are shown in Table 3. Supporting Hypotheses 1 and 2, team cooperative goals were significantly related to team time consensus (*b* = 0.453, *p* < 0.001), and team time consensus was significantly related to team thriving at work (*b* = 0.350, *p* < 0.001).

**Figure 2:**
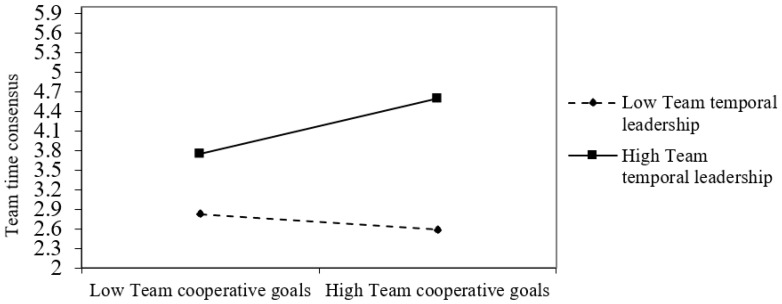
Moderating effect of temporal leadership between team cooperative goals and team time consensus.

As shown in Model 2 in Table 3, the interaction term of team cooperative goals and team temporal leadership in predicting team time consensus was significant (*b* = 0.271, *p* < 0.05). As depicted in Figure 2 and Table 4, simple slopes tests revealed that the relationship between team cooperative goals and team time consensus was significant and positive when team temporal leadership was high (*b* = 0.266, SE = 0.091, *p* < 0.01) but was not significant when team temporal leadership was low (*b* = 0.040, SE = 0.108, *p* > 0.05). The difference between these effects was also significant (Δ*b* = 0.226, 95% CI [0.062, 0.413]). These results provide support for Hypothesis 4a.

As in Table 4, with Monte Carlo simulation, the indirect effects through team time consensus were significant. The confidence intervals for the indirect effects via team time consensus did not contain zero (ab = 0.174, 95% CI [0.077, 0.323]). Thus, Hypothesis 3 received support.

The complete moderated mediation analysis results are reported in Table 5. The Monte Carlo results showed that the indirect effect of team cooperative goals on team thriving at work via team time consensus was significant when team temporal leadership was high (estimate = 0.075, 95% CI [0.011, 0.190]). However, the indirect effect was not significant when subordinate temporal leadership was low (estimate = 0.024, 95% CI [−0.022, 0.140]), and the difference between these indirect effects was also significant (Δ*b* = 0.050, 95% CI [0.003, 0.145]). Thus, Hypothesis 4b received support.

## 5. Discussion

From the perspective of time, this paper discusses the mechanism between team cooperative goals and team thriving at work, and the conclusions are as follows: (1) Team cooperative goals have a significant positive impact on team time consensus, and team time consensus has a significant positive impact on team thriving at work; (2) Team cooperative goals indirectly affect team thriving at work through team time consensus; and (3) Team temporal leadership not only moderates the relationship between team cooperative goals and team time consensus, but also moderates the indirect effect of team cooperative goals on team thriving at work through team time consensus.

### 5.1. Theoretical Implications

The theoretical significance of this paper is mainly reflected in the following aspects: First, previous studies usually use individual initiative behaviors, employees’ psychological states, and unit contextual features as a mediating mechanism to explore the emergence of thriving at work based on the socially embedded model [17,19]. Starting from the temporal perspective, the article explains the connective mechanism between team cooperative goals and team thriving at work, introduces team time consensus as a mediating variable, expands the previous theoretical research framework, and provides a new research perspective.

Second, while previous studies have mainly focused on thriving at work at the individual level, this paper focuses on thriving at work at the team level. The state of the entire team is closely related to team performance, and by focusing on team thriving at work, the article can be regarded as a useful supplement for previous research.

Third, this study proposes a moderated mediation model and examines the moderating role of temporal leadership. The previous literature lacked the test of the boundary conditions. This study examines the important factor of temporal leadership, which is helpful to enhance the understanding of the relationship between teamwork goals and thriving at work.

### 5.2. Practical Implications

The findings of this study also have some practical implications. First of all, the research results show that the team’s cooperative goals are conducive to the formation of team time consensus. The company management should actively set the team’s cooperative goals, weaken differences, form a good team atmosphere, promote team building, and strengthen team cohesion. Secondly, team time consensus mediates the relationship between team cooperative goals and thriving at work. The company should pay attention to the time orientation and rhythm pace of employees, strengthen the communication within the team, promote the team to reach a consensus on time, weaken the contradiction caused by time diversity, and promote the overall good state of the team. Finally, the research shows that the positive effect of team cooperative goals is more significant in the situation of high-level temporal leadership. The company should intensify the training of company team leaders’ temporal leadership ability, improve their time management level for themselves and teams, coordinate team time resources, synchronize work rhythm, stimulate team vitality and passion, and promote the development of teams and organizations.

### 5.3. Limitations and Directions for Future Research

This study also has some limitations. First of all, this study mainly adopts a cross-sectional survey to conduct research and cannot fully determine the causal relationship. Future research can add experimental methods to further improve the scientificity and accuracy of the research results. Secondly, although this paper explores the relationship between team cooperative goals and team thriving at work from the perspective of time, there may be other potential mediating variables and boundary conditions. Future research can further try other perspectives, such as emotional perspective. Finally, this study mainly focuses on the antecedents of thriving at work, while ignoring the impact of team cooperative goals on employee work outcomes. However, there are few studies on the effects of team cooperative goals. Future research can further explore whether team cooperative goals will affect employees’ work performance, creativity, and innovative behavior.

## 6. Conclusions

In this study, we examine the impact of team cooperative goals on team thriving at work from a time perspective and reveal their intrinsic mechanisms. The mediation effect of team time consensus and the moderation effect of team temporal leadership were identified. Through this study, we clearly realized that team cooperative goals are extremely important for mutual cooperation among team members, and that teams with cooperative goals are more likely to reach time consensus on task scheduling and further stimulate team thriving at work, which is meaningful for organizations. In a rapidly changing and intense market competition, where team members thriving at work is conducive to organizational innovation and development, organizations need to focus on the setting of internal cooperative goals. In addition, we found that under high-level team temporal leadership, team cooperative goals have a significant impact on time consensus, which in turn strengthens the positive impact on team thriving at work. The finding provides inspirations for managers on how to stimulate thriving at work among team members.

## Figures and Tables

**Table 1:** Results for Confirmatory Factor Analysis.

Model	χ^2^	Df	χ^2^/Df	CFI	TLI	SRMR
four-factor model (CG, TL, TC, TW)	284.734	59	4.826	0.897	0.864	0.054
three-factor model (CG + TL, TC, TW)	664.192	62	10.713	0.752	0.688	0.102
two-factor model (CG + TL, TC + TW)	878.455	64	13.726	0.664	0.591	0.125
one-factor model (CG + TL + TC + TW)	926.859	65	14.259	0.645	0.574	0.118

Note: Team cooperative goals (GC); Team temporal leadership (TL); Team time consensus (TC); Thriving at work (TW).

**Table 2:** Mean, Standard Deviation, and Correlation Coefficients of Variables.

Variable	M	SD	1	2	3	4	5	6	7	8	9
1. Age	34.88	5.80	1								
2. Gender	0.54	0.36	0.086	1							
3. Education	2.53	0.86	−0.527 ***	−0.155	1						
4. Years of working	6.55	3.41	0.478 ***	−0.228 *	−0.149	1					
5. Years of working with leaders	4.41	2.30	0.284 **	−0.220 *	−0.247 *	0.470 ***	1				
6. Team cooperative goals	4.07	0.47	−0.172	0.090	0.329 ***	−0.198	0.073	1			
7. Team temporal leadership	4.03	0.42	−0.072	0.204	0.114	−0.261 *	−0.219 *	0.449 ***	1		
8. Team time consensus	3.94	0.47	0.005	0.266 *	0.109	−0.221 *	−0.094	0.473 ***	0.767 ***	1	
9. Team thriving at work	3.66	0.38	0.180	0.332 ***	−0.121	0.027	−0.066	0.109	0.393 ***	0.453 ***	1

Note. * *p* < 0.05, ** *p* < 0.01, *** *p* < 0.001.

**Table 3:** Results for Regression Analysis.

Variable	Team Time Consensus		Team Thriving at Work		
	Model 1	Model 2	Model 3	Model 4	Model 5
Intercept	1.595 **	3.436 ***	3.177 ***	2.133 ***	2.204 ***
Age	0.014	0.009	0.009	0.003	0.003
Gender	0.229	0.176 *	0.339 ***	0.226 **	0.229 **
Education	0.024	0.019	−0.002	−0.049	−0.042
Years of working	−0.020	−0.011	0.007	0.020	0.019
Years of working with leaders	−0.013	0.023	−0.011	−0.017	−0.015
Team cooperative goals	0.453 ***	0.153			−0.038
Team temporal leadership		0.734 ***			
Team cooperative goals × Team temporal leadership		0.271 *			
Team time consensus				0.350 ***	0.366 ***
R^2^	0.302	0.654	0.137	0.303	0.274 **

Note. * *p* < 0.05, ** *p* < 0.01, *** *p* < 0.001.

**Table 4:** Results for Mediating and Moderating Effect.

		b	SE	95% CI	
				Lower	Upper
Mediating Role	Team time consensus	0.174	0.061	0.077	0.323
Moderating Role	High team temporal leadership (M + 1SD)	0.266 **	0.091	0.102	0.467
	Low team temporal leadership (M − 1SD)	0.040	0.108	−0.146	0.277
	Diff	0.226 *	0.091	0.062	0.413

Note. * *p* < 0.05, ** *p* < 0.01.

**Table 5:** Test Results for Moderated Mediation Effects.

	b	SE	95%CI	
			Lower	Upper
High (M + 1SD)	0.075	0.044	0.011	0.190
Low (M − 1SD)	0.024	0.037	−0.022	0.140
Diff	0.050	0.034	0.003	0.145

## Data Availability

Not applicable.
